# The genome sequence of the club-tailed millipede, *Cylindroiulus punctatus *(Leach, 1816)

**DOI:** 10.12688/wellcomeopenres.24251.1

**Published:** 2025-05-20

**Authors:** Gregory D. Edgecombe, Liam M. Crowley, Mark G. Telfer, University of Oxford and Wytham Woods Genome Acquisiiton Lab, Darwin Tree of Life Barcoding collective, Wellcome Sanger Institute Tree of Life Management, Samples and Laboratory team, Wellcome Sanger Institute Scientific Operations: Sequencing Operations, Wellcome Sanger Institute Tree of Life Core Informatics team, Tree of Life Core Informatics collective, Darwin Tree of Life Consortium

**Affiliations:** 1 Natural History Museum, London, England, UK; 2 University of Oxford, Oxford, England, UK; 3 Independent researcher, Ventnor, Isle of Wight, UK

**Keywords:** Cylindroiulus punctatus, club-tailed millipede, genome sequence, chromosomal, Julida

## Abstract

We present a genome assembly from a female specimen of *Cylindroiulus punctatus* (club-tailed millipede; Arthropoda; Diplopoda; Julida; Julidae). The genome sequence has a total length of 354.09 megabases. Most of the assembly (92.9%) is scaffolded into 7 chromosomal pseudomolecules. The mitochondrial genome has also been assembled, with a length of 18.98 kilobases.

## Species taxonomy

Eukaryota; Opisthokonta; Metazoa; Eumetazoa; Bilateria; Protostomia; Ecdysozoa; Panarthropoda; Arthropoda; Mandibulata; Myriapoda; Diplopoda; Helminthomorpha; Julida; Julidae; *Cylindroiulus*; *Cylindroiulus punctatus* (Leach, 1816) (NCBI:txid61981)

## Background

*Cylindroiulus punctatus* is widespread throughout western Europe, ranging from southern Scandinavia and the Shetlands to northern Spain and the Balearics ( Kime & Enghoff, 2017). It has been introduced into Newfoundland and Nova Scotia, Canada ( Shelley & Smith, 2011). It is the most commonly recorded millipede in Britain and Ireland, found in nearly all parts of both islands ( Lee, 2006).

Occurrence data follow Lee (2006) and Kime and Enghoff (2017). *Cylindroiulus punctatus* is strongly associated with woodland, and in Britain and Ireland has a negative association with cultivation, sand dunes and grassland. It is commonest in dead and decaying wood and under the bark of dead trees, and it feeds on both wood and leaf litter. Seasonal vertical migration involves overwintering in soil and shifting to litter and then logs during the warmer months, when mating and oviposition occur ( Banerjee, 1967); by spring it can be found in trees and may be metres above ground in summer ( Kime & Enghoff, 2017). It is generally a thermophilic lowland species, though exceeding 1000 m in its southern limit in northeastern Spain. In the British Isles, its distribution is rural and semi-natural more so than synanthropic/urban, and it is associated with non-calcareous, commonly loamy soils.

Its common name refers to a club-shaped caudal projection in adults and later immature stadia ( Blower, 1985), otherwise seen only in the less common *C. londinensis* (Leach, 1816) among the 13 British species of *Cylindroiulus* (bmig.org.uk/checklist/millipede-checklist). The two are distinguished by colour and size, *C. punctatus* being straw- or pinkish brown and up to 28 mm long in females (20 mm in males) and *C. londinensis* being darker (nearly black) and of length to 48 mm. Anatomical and developmental data are relatively well known, the former including the fine structure of sensilla ( Nguyen Duy-Jacquemin, 1990) and composition of defensive secretions ( Huth, 2000). Mating behaviour has been documented ( Haacker & Fuchs, 1970). Nests are made beneath bark of fallen logs and stumps, found in the field in Cheshire from May to August ( Blower & Gabbutt, 1964). Batches of eggs number 45 to 60 ( Saudray, 1952) and the duration of post-embryonic stadia has been documented in populations from France ( Saudray, 1952) and oak woodland in Devon ( Blower & Gabbutt, 1964). In the latter, maturity is reached at stadium VIII during the animal’s third winter, with females able to breed for several additional years to a maximum of 14 stadia. 

Few whole genome sequences for millipedes have been generated, these consisting of *Trigonoiulus corallinus* (GCA_013389805.1 ( Kenny *et al.*, 2015), *Helicorthomorpha holstii* (GCA_013389785.1) ( Qu *et al.*, 2020), and of *Glomeris maerens* (GCA_023279145.1), *Anaulaciulus tonginus* (GCA_023279205.1) and *Niponia nodulosa* (GCA_023279205.1). ( So *et al.*, 2022). The MetaInvert database at Senckenberg Görlitz and the LOEWE Centre for Translational Biodiversity Genomics provide genome sequences for many soil invertebrates, among them 23 Diplopoda, including *Cylindroiulus punctatus* ( Collins *et al.*, 2023).

**Figure 1.**
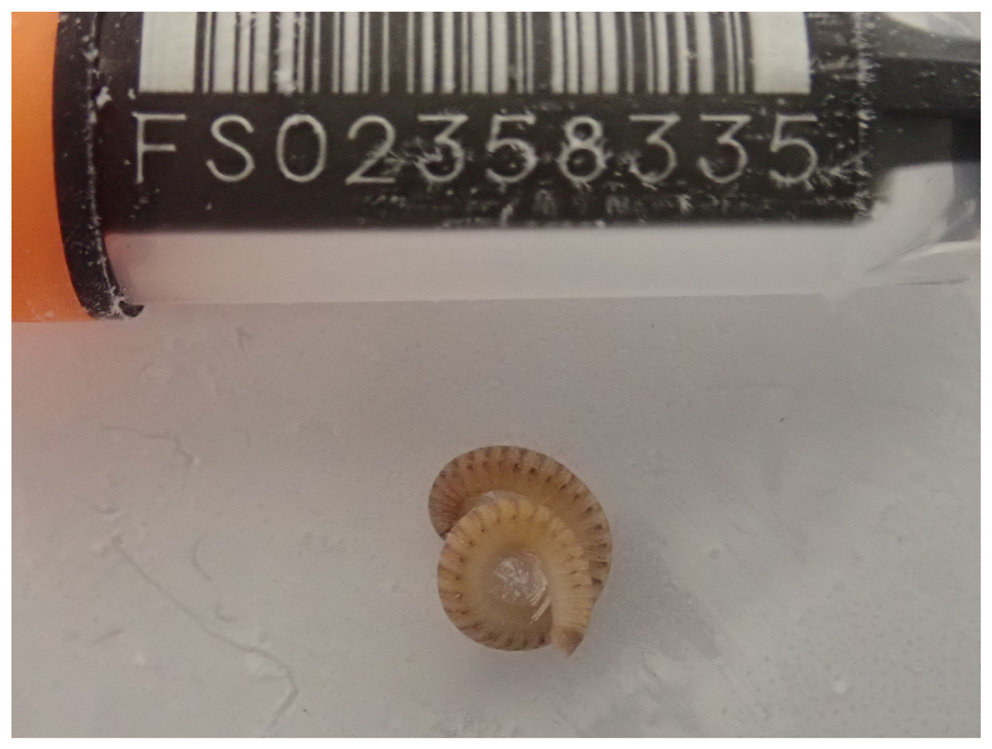
Photograph of the *Cylindroiulus punctatus* (qdCylPunc2) specimen used for genome sequencing.

The genome of *Cylindroiulus punctatus* ( Figure 1) was sequenced as part of the Darwin Tree of Life Project, a collaborative effort to sequence all named eukaryotic species in the Atlantic Archipelago of Britain and Ireland. Here we present a chromosomally complete genome sequence for *Cylindroiulus punctatus*, based on one adult specimen from Wytham Woods, Oxfordshire, England (latitude 51.772, longitude –1.338). The genome is of interest because the species is the most common and widespread British millipede.

## Genome sequence report

### Sequencing data

The genome of a specimen of *Cylindroiulus punctatus* ( Figure 1) was sequenced using Pacific Biosciences single-molecule HiFi long reads, generating 20.76 Gb (gigabases) from 2.19 million reads. GenomeScope analysis of the PacBio HiFi data estimated the haploid genome size at 332.51 Mb, with a heterozygosity of 0.60% and repeat content of 37.34%. These values provide an initial assessment of genome complexity and the challenges anticipated during assembly. Based on this estimated genome size, the sequencing data provided approximately 55 coverage of the genome. Chromosome conformation Hi-C sequencing produced 145.82 Gb from 965.70 million reads. Table 1 summarises the specimen and sequencing information.

**Table 1.** Specimen and sequencing data for *Cylindroiulus punctatus*.

Project information			
**Study title**	Cylindroiulus punctatus		
**Umbrella BioProject**	PRJEB68093		
**Species**	*Cylindroiulus punctatus*		
**BioSpecimen**	SAMEA8603225		
**NCBI taxonomy ID**	61981		
Specimen information			
**Technology**	**ToLID**	**BioSample accession**	**Organism part**
**PacBio long read sequencing**	qdCylPunc2	SAMEA8603818	whole organism
**Hi-C sequencing**	qdCylPunc1	SAMEA8603815	whole organism
**RNA sequencing**	qdCylPunc4	SAMEA9066141	mid-body
Sequencing information			
**Platform**	**Run accession**	**Read count**	**Base count (Gb)**
**Hi-C Illumina NovaSeq 6000**	ERR12245646	9.66e+08	145.82
**PacBio Sequel IIe**	ERR12205309	2.19e+06	20.76
**RNA Illumina HiSeq 4000**	ERR12245647	6.05e+07	9.13

### Assembly statistics

The primary haplotype was assembled, and contigs corresponding to an alternate haplotype were also deposited in INSDC databases. The assembly was improved by manual curation, which corrected 136 misjoins or missing joins. These interventions decreased the scaffold count by 20.31% and increased the scaffold N50 by 201.73%. The final assembly has a total length of 354.09 Mb in 207 scaffolds, with 385 gaps, and a scaffold N50 of 47.05 Mb ( Table 2).

**Table 2.** Genome assembly data for *Cylindroiulus punctatus*.

Genome assembly		
Assembly name	qdCylPunc2.1	
Assembly accession	GCA_965125795.1	
*Alternate haplotype accession*	*GCA_965125805.1*	
Assembly level for primary assembly	chromosome	
Span (Mb)	354.09	
Number of contigs	592	
Number of scaffolds	207	
Longest scaffold (Mb)	63.47	
Assembly metric	Measure	*Benchmark*
Contig N50 length	1.37 Mb	*≥ 1 Mb*
Scaffold N50 length	47.05 Mb	*= chromosome N50*
Consensus quality (QV)	Primary: 63.3; alternate: 63.1; combined: 63.2	*≥ 40*
*k*-mer completeness	Primary: 87.16%; alternate: 55.02%; combined: 97.66%	*≥ 95%*
BUSCO *	C:97.0%[S:95.8%,D:1.3%], F:2.0%,M:1.0%,n:1,013	*S > 90%; D < 5%*
Percentage of assembly mapped to chromosomes	92.9%	*≥ 90%*
Sex chromosomes	Not identified	*localised homologous pairs*
Organelles	Mitochondrial genome: 18.98 kb	*complete single alleles*

* BUSCO scores based on the arthropoda_odb10 BUSCO set using version 5.5.0. C = complete [S = single copy, D = duplicated], F = fragmented, M = missing, n = number of orthologues in comparison.

**Figure 2.**
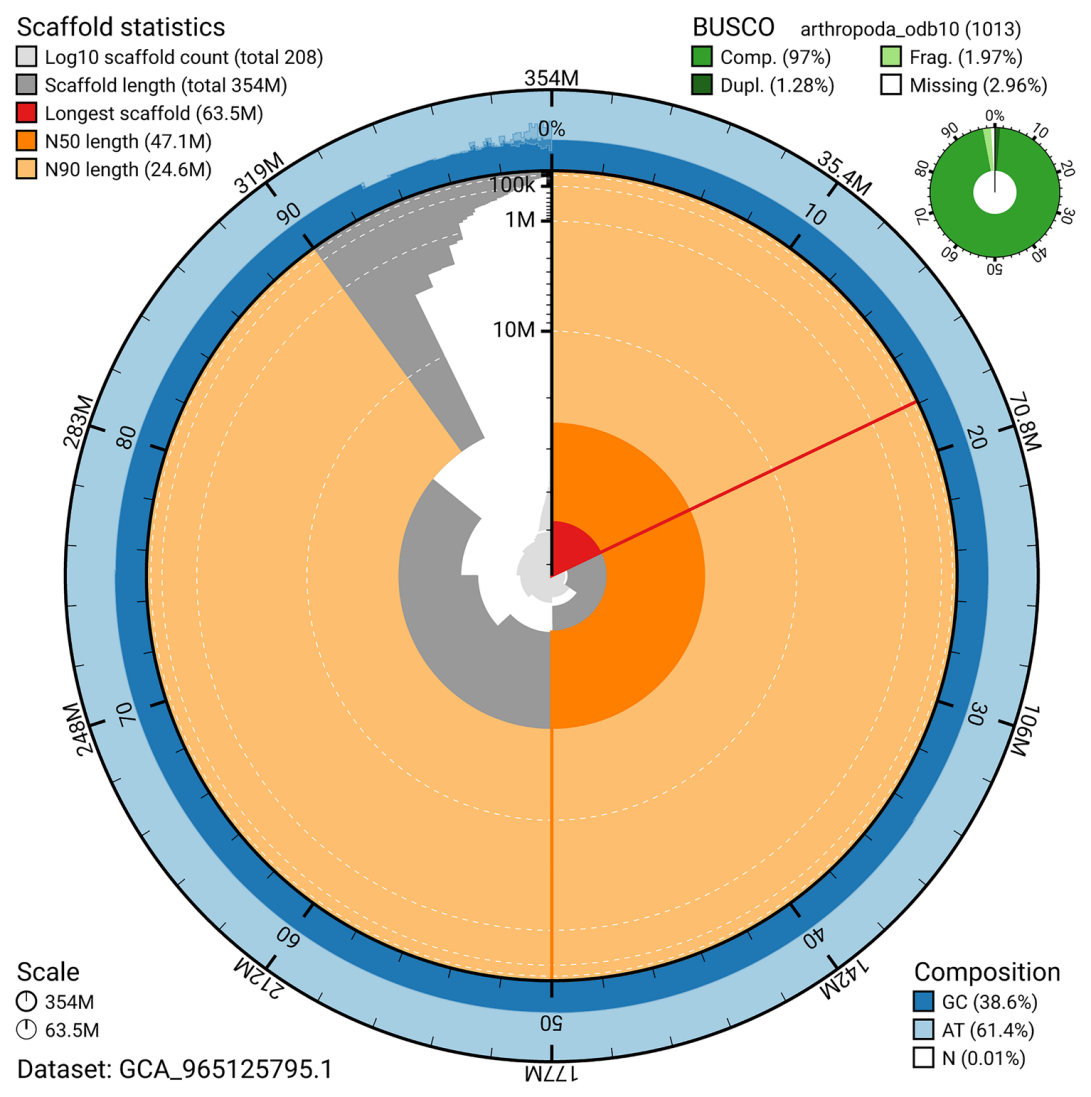
Genome assembly of *Cylindroiulus punctatus*, qdCylPunc2.1: metrics. The BlobToolKit snail plot provides an overview of assembly metrics and BUSCO gene completeness. The circumference represents the length of the whole genome sequence, and the main plot is divided into 1,000 bins around the circumference. The outermost blue tracks display the distribution of GC, AT, and N percentages across the bins. Scaffolds are arranged clockwise from longest to shortest and are depicted in dark grey. The longest scaffold is indicated by the red arc, and the deeper orange and pale orange arcs represent the N50 and N90 lengths. A light grey spiral at the centre shows the cumulative scaffold count on a logarithmic scale. A summary of complete, fragmented, duplicated, and missing BUSCO genes in the arthropoda_odb10 set is presented at the top right. An interactive version of this figure is available at https://blobtoolkit.genomehubs.org/view/GCA_965125795.1/dataset/GCA_965125795.1/snail.

**Figure 3.**
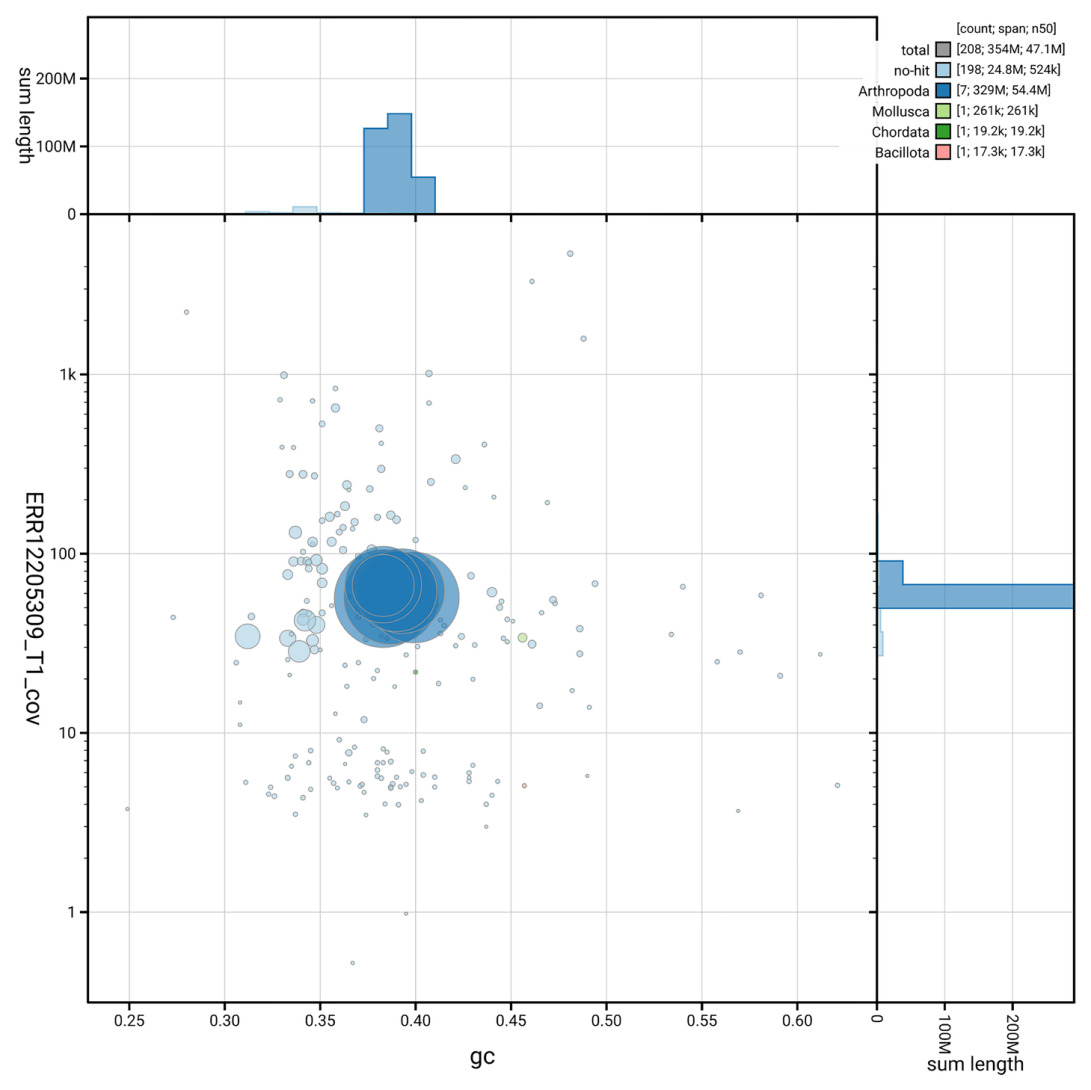
Genome assembly of *Cylindroiulus punctatus*, qdCylPunc2.1: BlobToolKit GC-coverage plot. Blob plot showing sequence coverage (vertical axis) and GC content (horizontal axis). The circles represent scaffolds, with the size proportional to scaffold length and the colour representing phylum membership. The histograms along the axes display the total length of sequences distributed across different levels of coverage and GC content. An interactive version of this figure is available at https://blobtoolkit.genomehubs.org/view/GCA_965125795.1/dataset/GCA_965125795.1/blob.

**Figure 4.**
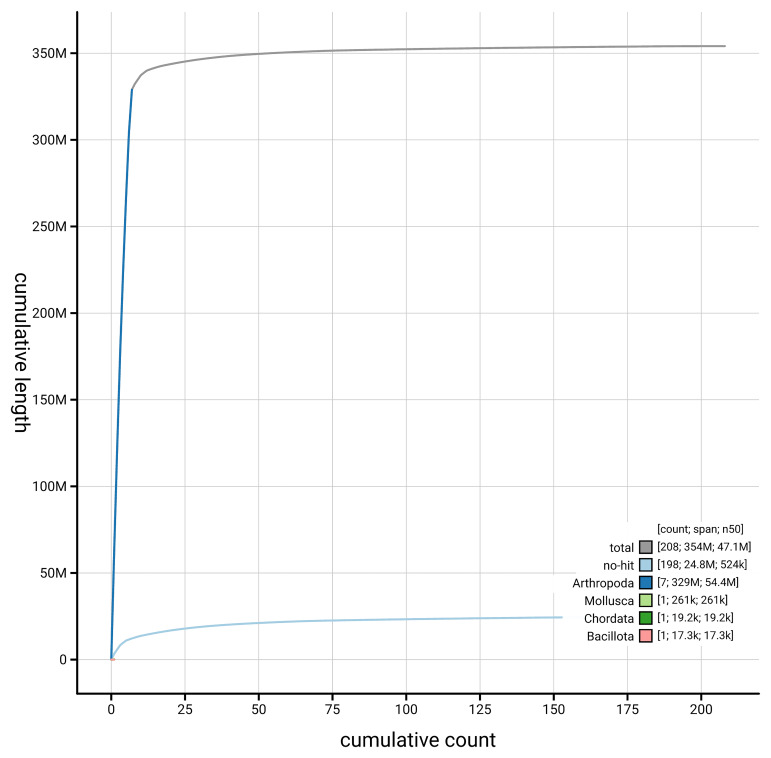
Genome assembly of *Cylindroiulus punctatus,* qdCylPunc2.1: BlobToolKit cumulative sequence plot. The grey line shows cumulative length for all scaffolds. Coloured lines show cumulative lengths of scaffolds assigned to each phylum using the buscogenes taxrule. An interactive version of this figure is available at https://blobtoolkit.genomehubs.org/view/GCA_965125795.1/dataset/GCA_965125795.1/cumulative.

The snail plot in Figure 2 provides a summary of the assembly statistics, indicating the distribution of scaffold lengths and other assembly metrics. Figure 3 shows the distribution of scaffolds by GC proportion and coverage. Figure 4 presents a cumulative assembly plot, with separate curves representing different scaffold subsets assigned to various phyla, illustrating the completeness of the assembly.

**Figure 5.**
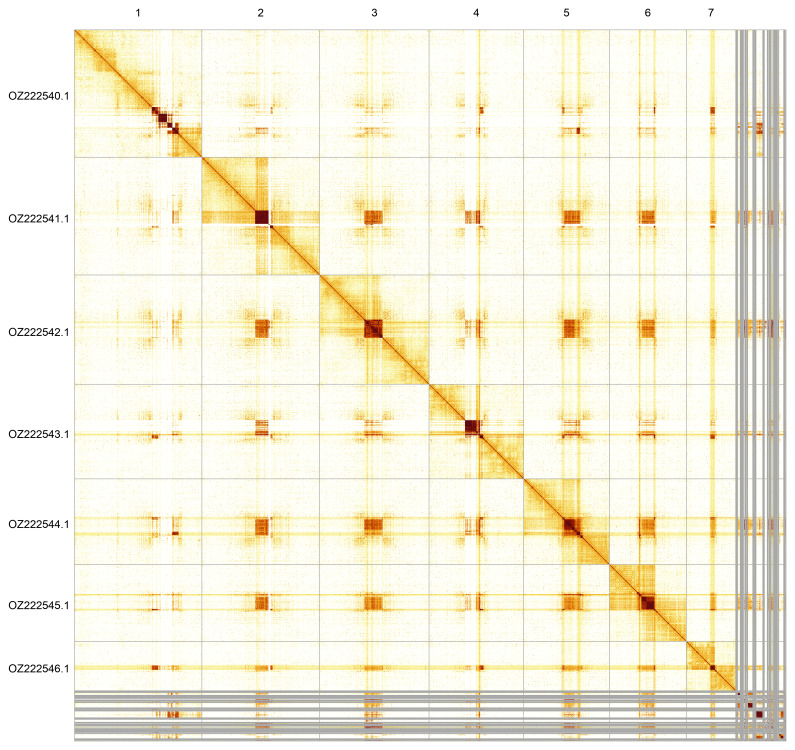
Genome assembly of *Cylindroiulus punctatus*: Hi-C contact map of the qdCylPunc2.1 assembly, produced in PretextView. Chromosomes are shown in order of size from left to right and top to bottom.

Most of the assembly sequence (92.9%) was assigned to 7 chromosomal-level scaffolds. These chromosome-level scaffolds, confirmed by Hi-C data, are named according to size ( Figure 5; Table 3).

**Table 3.** Chromosomal pseudomolecules in the genome assembly of *Cylindroiulus punctatus*, qdCylPunc2.

INSDC accession	Name	Length (Mb)	GC%
OZ222540.1	1	63.47	38.5
OZ222541.1	2	58.63	38.5
OZ222542.1	3	54.39	40
OZ222543.1	4	47.05	39.5
OZ222544.1	5	42.55	39
OZ222545.1	6	38.3	38.5
OZ222546.1	7	24.58	38.5
OZ222547.1	MT	0.02	28

The mitochondrial genome was also assembled. This sequence is included as a contig in the multifasta file of the genome submission and as a standalone record.

### Assembly quality metrics

The estimated Quality Value (QV) and *k*-mer completeness metrics, along with BUSCO completeness scores, were calculated for each haplotype and the combined assembly. The QV reflects the base-level accuracy of the assembly, while *k*-mer completeness indicates the proportion of expected *k*-mers identified in the assembly. BUSCO scores provide a measure of completeness based on benchmarking universal single-copy orthologues.

The combined primary and alternate assemblies achieve an estimated QV of 63.2. The *k*-mer recovery for the primary haplotype is 87.16%, and for the alternate haplotype 55.02%; the combined primary and alternate assemblies have a *k*-mer recovery of 97.66%. BUSCO v.5.5.0 analysis using the arthropoda_odb10 reference set ( *n* = 1,013) identified 97.0% of the expected gene set (single = 95.8%, duplicated = 1.3%).

Table 2 provides assembly metric benchmarks adapted from Rhie *et al.* (2021) and the Earth BioGenome Project Report on Assembly Standards September 2024. The primary assembly achieves the EBP reference standard of **6.C.Q63**.

## Methods

### Sample acquisition and DNA barcoding

*Cylindroiulus punctatus* specimens were collected from Wytham Woods, Oxfordshire, UK (latitude 51.772, longitude –1.338) on 2020-12-08 by potting. The specimens were collected by Liam Crowley (University of Oxford) and identified by Mark Telfer (University of Oxford) and preserved on dry ice. One specimen (specimen ID Ox001002, ToLID qdCylPunc2) was used for PacBio HiFi sequencing and another (specimen ID Ox001001, ToLID qdCylPunc1) was used for Hi-C sequencing.

The specimen used for RNA sequencing (specimen ID NHMUK014449163, ToLID qdCylPunc4) was collected from Barnes Common, England, UK (latitude 51.467, longitude –0.235) on 2020-11-19 by handpicking. The specimen was collected and identified by Gregory Edgecombe (Natural History Museum) and dry frozen.

The initial identification was verified by an additional DNA barcoding process according to the framework developed by Twyford *et al*. (2024). A small sample was dissected from the specimen and stored in ethanol, while the remaining parts were shipped on dry ice to the Wellcome Sanger Institute (WSI) ( Pereira *et al.*, 2022). The tissue was lysed, the COI marker region was amplified by PCR, and amplicons were sequenced and compared to the BOLD database, confirming the species identification ( Crowley *et al.*, 2023). Following whole genome sequence generation, the relevant DNA barcode region was also used alongside the initial barcoding data for sample tracking at the WSI ( Twyford *et al.*, 2024). The standard operating procedures for Darwin Tree of Life barcoding have been deposited on protocols.io ( Beasley *et al.*, 2023).

Metadata collection for samples adhered to the Darwin Tree of Life project standards described by Lawniczak *et al*. (2022).

### Nucleic acid extraction

The workflow for high molecular weight (HMW) DNA extraction at the Wellcome Sanger Institute (WSI) Tree of Life Core Laboratory includes a sequence of procedures: sample preparation and homogenisation, DNA extraction, fragmentation and purification. Detailed protocols are available on protocols.io ( Denton *et al.*, 2023b).

The qdCylPunc2 sample was prepared for DNA extraction by weighing and dissecting it on dry ice ( Jay *et al.*, 2023). Tissue from the whole organism was homogenised using a PowerMasher II tissue disruptor ( Denton *et al.*, 2023a). HMW DNA was extracted using the Automated MagAttract v1 protocol ( Sheerin *et al.*, 2023). For ultra-low input (ULI) PacBio sequencing, DNA was fragmented using the Covaris g-TUBE method ( Oatley *et al.*, 2023). Sheared DNA was purified by solid-phase reversible immobilisation, using AMPure PB beads to eliminate shorter fragments and concentrate the DNA ( Strickland *et al.*, 2023). The concentration of the sheared and purified DNA was assessed using a Nanodrop spectrophotometer and a Qubit Fluorometer using the Qubit dsDNA High Sensitivity Assay kit. The fragment size distribution was evaluated by running the sample on the FemtoPulse system.

RNA was extracted from mid-body tissue of qdCylPunc4 in the Tree of Life Laboratory at the WSI using the RNA Extraction: Automated MagMax™ *mir*Vana protocol ( do Amaral *et al.*, 2023). The RNA concentration was assessed using a Nanodrop spectrophotometer and a Qubit Fluorometer using the Qubit RNA Broad-Range Assay kit. Analysis of the integrity of the RNA was done using the Agilent RNA 6000 Pico Kit and Eukaryotic Total RNA assay.

### DNA library preparation and sequencing

Library preparation and sequencing were performed at the WSI Scientific Operations core.

***PacBio HiFi***

The input samples for ultra-low input (ULI) library preparation were sheared to approximately 10 kb using the Covaris g-TUBE. ULI libraries were prepared using the PacBio SMRTbell Express Template Prep Kit 2.0 and SMRTbell gDNA Sample Amplification Kit (Pacific Biosciences, California, USA). DNA was normalised to 20 ng before initial removal of single-strand overhangs, DNA damage repair, and end repair/A-tailing, following the manufacturer’s instructions. Amplification adapters were ligated using the SMRTbell gDNA Sample Amplification Kit. A 0.85× pre-PCR cleanup was performed with Promega ProNex beads, and the sample was split for dual PCR (PCR reactions A and B), each following the manufacturer’s protocol. After amplification, both PCR reactions underwent a 0.85× post-PCR cleanup with ProNex beads. DNA concentration was quantified using a Qubit Fluorometer v2.0 (Thermo Fisher Scientific) with the Qubit HS Assay Kit, and fragment size was analysed on an Agilent Femto Pulse Automated Pulsed Field CE Instrument (Agilent Technologies) using the gDNA 55 kb BAC analysis kit. PCR reactions A and B were then pooled to achieve a total mass of ≥500 ng in 47.4 μl. The pooled sample underwent DNA damage repair, end repair/A-tailing, and additional hairpin adapter ligation before a 1× cleanup with ProNex beads. DNA concentration and fragment size were reassessed using Qubit and Femto Pulse analysis. Size selection was performed using a Sage Science PippinHT system, with target fragment size determined from the Femto Pulse analysis, typically between 4000 and 9000 bp. Size-selected libraries underwent a final 1.0× cleanup with ProNex beads and were normalised to 2 nM before sequencing.

Samples were sequenced using the Sequel IIe system (Pacific Biosciences, California, USA). The concentration of the library loaded onto the Sequel IIe was in the range 40–135 pM. The SMRT link software, a PacBio web-based end-to-end workflow manager, was used to set-up and monitor the run, as well as perform primary and secondary analysis of the data upon completion.

### Hi-C

***Sample preparation and crosslinking***

Hi-C samples were prepared from 20–50 mg of frozen tissue from the qdCylPunc1 sample using the Arima-HiC v2 kit (Arima Genomics). Following the manufacturer’s instructions, tissue was fixed and DNA crosslinked using TC buffer, with a final formaldehyde concentration of 2%. The tissue was homogenised using the Diagnocine Power Masher-II. Crosslinked DNA was digested with a restriction enzyme master mix, biotinylated, and ligated. Clean-up was performed with SPRISelect beads before library preparation. DNA concentration was measured with the Qubit Fluorometer (Thermo Fisher Scientific) and Qubit HS Assay Kit. The biotinylation percentage was estimated using the Arima-HiC v2 QC beads.

***Hi-C library preparation, amplification and sequencing***

Biotinylated DNA constructs were fragmented using a Covaris E220 sonicator and size selected to 400–600 bp using SPRISelect beads. DNA was enriched with Arima-HiC v2 kit Enrichment beads. End repair, A-tailing, and adapter ligation were carried out with the NEBNext Ultra II DNA Library Prep Kit (New England Biolabs), following a modified protocol where library preparation occurs while DNA remains bound to the Enrichment beads. Library amplification was performed using KAPA HiFi HotStart mix and a custom Unique Dual Index (UDI) barcode set (Integrated DNA Technologies). Depending on sample concentration and biotinylation percentage determined at the crosslinking stage, libraries were amplified with 10–16 PCR cycles. Post-PCR clean-up was performed with SPRISelect beads. Libraries were quantified using the AccuClear Ultra High Sensitivity dsDNA Standards Assay Kit (Biotium) and a FLUOstar Omega plate reader (BMG Labtech).

Prior to sequencing, libraries were normalised to 10 ng/μL. Normalised libraries were quantified again (as above) and used to create equimolar and/or weighted 2.8 nM pools. Pool concentrations were checked using the Agilent 4200 TapeStation (Agilent) with High Sensitivity D500 reagents before sequencing. Sequencing was performed using paired-end 150 bp reads on the Illumina NovaSeq 6000.

***RNA***

Libraries were prepared using the NEBNext ^®^ Ultra™ II Directional RNA Library Prep Kit for Illumina (New England Biolabs), following the manufacturer’s instructions. Poly(A) mRNA in the total RNA solution was isolated using oligo(dT) beads, converted to cDNA, and uniquely indexed; 14 PCR cycles were performed. Libraries were size-selected to produce fragments between 100–300 bp. Libraries were quantified, normalised, pooled to a final concentration of 2.8 nM, and diluted to 150 pM for loading. Sequencing was carried out on the Illumina HiSeq 4000 to generate 150-bp paired-end reads.

### Genome assembly, curation and evaluation

***Assembly***

Prior to assembly of the PacBio HiFi reads, a database of *k*-mer counts ( *k* = 31) was generated from the filtered reads using FastK. GenomeScope2 ( Ranallo-Benavidez *et al.*, 2020) was used to analyse the *k*-mer frequency distributions, providing estimates of genome size, heterozygosity, and repeat content.

The HiFi reads were first assembled using Hifiasm ( Cheng *et al.*, 2021) with the --primary option. Haplotypic duplications were identified and removed using purge_dups ( Guan *et al.*, 2020). The Hi-C reads ( Rao *et al.*, 2014) were mapped to the primary contigs using bwa-mem2 ( Vasimuddin *et al.*, 2019), and the contigs were scaffolded using YaHS ( Zhou *et al.*, 2023) using the --break option for handling potential misassemblies. The scaffolded assemblies were evaluated using Gfastats ( Formenti *et al.*, 2022), BUSCO ( Manni *et al.*, 2021) and MERQURY.FK ( Rhie *et al.*, 2020).

The mitochondrial genome was assembled using MitoHiFi ( Uliano-Silva *et al.*, 2023), which runs MitoFinder ( Allio *et al.*, 2020) and uses these annotations to select the final mitochondrial contig and to ensure the general quality of the sequence.

***Assembly curation***

The assembly was decontaminated using the Assembly Screen for Cobionts and Contaminants (ASCC) pipeline. Flat files and maps used in curation were generated via the TreeVal pipeline ( Pointon *et al.*, 2023). Manual curation was conducted primarily in PretextView ( Harry, 2022) and HiGlass ( Kerpedjiev *et al.*, 2018), with additional insights provided by JBrowse2 ( Diesh *et al.*, 2023). Scaffolds were visually inspected and corrected as described by Howe *et al*. (2021). Any identified contamination, missed joins, and mis-joins were amended, and duplicate sequences were tagged and removed. The curation process is documented at https://gitlab.com/wtsi-grit/rapid-curation.

***Assembly quality assessment***

The Merqury.FK tool ( Rhie *et al.*, 2020), run in a Singularity container ( Kurtzer *et al.*, 2017), was used to evaluate *k*-mer completeness and assembly quality for the primary and alternate haplotypes using the *k*-mer databases ( *k* = 31) computed prior to genome assembly. The analysis outputs included assembly QV scores and completeness statistics.

The blobtoolkit pipeline is a Nextflow ( Di Tommaso *et al.*, 2017) port of the previous Snakemake Blobtoolkit pipeline ( Challis *et al.*, 2020). It aligns the PacBio reads in SAMtools and minimap2 ( Li, 2018) and generates coverage tracks for regions of fixed size. In parallel, it queries the GoaT database ( Challis *et al.*, 2023) to identify all matching BUSCO lineages to run BUSCO ( Manni *et al.*, 2021). For the three domain-level BUSCO lineages, the pipeline aligns the BUSCO genes to the UniProt Reference Proteomes database ( Bateman *et al.*, 2023) with DIAMOND blastp ( Buchfink *et al.*, 2021). The genome is also divided into chunks according to the density of the BUSCO genes from the closest taxonomic lineage, and each chunk is aligned to the UniProt Reference Proteomes database using DIAMOND blastx. Genome sequences without a hit are chunked using seqtk and aligned to the NT database with blastn ( Altschul *et al.*, 1990). The blobtools suite combines all these outputs into a blobdir for visualisation.

The blobtoolkit pipeline was developed using nf-core tooling ( Ewels *et al.*, 2020) and MultiQC ( Ewels *et al.*, 2016), relying on the Conda package manager, the Bioconda initiative ( Grüning *et al.*, 2018), the Biocontainers infrastructure ( da Veiga Leprevost *et al.*, 2017), as well as the Docker ( Merkel, 2014) and Singularity ( Kurtzer *et al.*, 2017) containerisation solutions.

Table 4 contains a list of relevant software tool versions and sources. The Tree of Life pipelines can be accessed via this page: https://pipelines.tol.sanger.ac.uk/pipelines.

**Table 4.** Software tools: versions and sources.

Software tool	Version	Source
BLAST	2.14.0	ftp://ftp.ncbi.nlm.nih.gov/blast/executables/blast+/
BlobToolKit	4.3.9	https://github.com/blobtoolkit/blobtoolkit
BUSCO	5.5.0	https://gitlab.com/ezlab/busco
bwa-mem2	2.2.1	https://github.com/bwa-mem2/bwa-mem2
DIAMOND	2.1.8	https://github.com/bbuchfink/diamond
fasta_windows	0.2.4	https://github.com/tolkit/fasta_windows
FastK	666652151335353eef2fcd58880bcef5bc2928e1	https://github.com/thegenemyers/FASTK
Gfastats	1.3.6	https://github.com/vgl-hub/gfastats
GoaT CLI	0.2.5	https://github.com/genomehubs/goat-cli
Hifiasm	0.16.1	https://github.com/chhylp123/hifiasm
HiGlass	44086069ee7d4d3f6f3f0012569789ec138f42b84aa44357826c0b6753eb28de	https://github.com/higlass/higlass
MerquryFK	d00d98157618f4e8d1a9190026b19b471055b22e	https://github.com/thegenemyers/MERQURY.FK
Minimap2	2.24-r1122	https://github.com/lh3/minimap2
MitoHiFi	2	https://github.com/marcelauliano/MitoHiFi
MultiQC	1.14, 1.17, and 1.18	https://github.com/MultiQC/MultiQC
Nextflow	23.10.0	https://github.com/nextflow-io/nextflow
PretextView	0.2.5	https://github.com/sanger-tol/PretextView
purge_dups	1.2.3	https://github.com/dfguan/purge_dups
sanger-tol/ascc	-	https://github.com/sanger-tol/ascc
sanger-tol/blobtoolkit	0.6.0	https://github.com/sanger-tol/blobtoolkit
Seqtk	1.3	https://github.com/lh3/seqtk
Singularity	3.9.0	https://github.com/sylabs/singularity
TreeVal	1.2.0	https://github.com/sanger-tol/treeval
YaHS	1.1a.2	https://github.com/c-zhou/yahs

### Wellcome Sanger Institute – Legal and Governance

The materials that have contributed to this genome note have been supplied by a Darwin Tree of Life Partner. The submission of materials by a Darwin Tree of Life Partner is subject to the **‘Darwin Tree of Life Project Sampling Code of Practice’**, which can be found in full on the Darwin Tree of Life website here. By agreeing with and signing up to the Sampling Code of Practice, the Darwin Tree of Life Partner agrees they will meet the legal and ethical requirements and standards set out within this document in respect of all samples acquired for, and supplied to, the Darwin Tree of Life Project.

Further, the Wellcome Sanger Institute employs a process whereby due diligence is carried out proportionate to the nature of the materials themselves, and the circumstances under which they have been/are to be collected and provided for use. The purpose of this is to address and mitigate any potential legal and/or ethical implications of receipt and use of the materials as part of the research project, and to ensure that in doing so we align with best practice wherever possible. The overarching areas of consideration are:

•   Ethical review of provenance and sourcing of the material

•   Legality of collection, transfer and use (national and international)

Each transfer of samples is further undertaken according to a Research Collaboration Agreement or Material Transfer Agreement entered into by the Darwin Tree of Life Partner, Genome Research Limited (operating as the Wellcome Sanger Institute), and in some circumstances other Darwin Tree of Life collaborators.

## Data Availability

European Nucleotide Archive: Cylindroiulus punctatus. Accession number PRJEB68093; https://identifiers.org/ena.embl/PRJEB68093. The genome sequence is released openly for reuse. The *Cylindroiulus punctatus* genome sequencing initiative is part of the Darwin Tree of Life Project (PRJEB40665). All raw sequence data and the assembly have been deposited in INSDC databases. The genome will be annotated using available RNA-Seq data and presented through the Ensembl pipeline at the European Bioinformatics Institute. Raw data and assembly accession identifiers are reported in Table 1 and Table 2.

## Author information

Members of the University of Oxford and Wytham Woods Genome Acquisition Lab are listed here: https://doi.org/10.5281/zenodo.12157525.

Members of the Natural History Museum Genome Acquisition Lab are listed here: https://doi.org/10.5281/zenodo.12159242.

Members of the Darwin Tree of Life Barcoding collective are listed here: https://doi.org/10.5281/zenodo.12158331.

Members of the Wellcome Sanger Institute Tree of Life Management, Samples and Laboratory team are listed here: https://doi.org/10.5281/zenodo.12162482.

Members of Wellcome Sanger Institute Scientific Operations: Sequencing Operations are listed here: https://doi.org/10.5281/zenodo.12165051.

Members of the Wellcome Sanger Institute Tree of Life Core Informatics team are listed here: https://doi.org/10.5281/zenodo.12160324.

Members of the Tree of Life Core Informatics collective are listed here: https://doi.org/10.5281/zenodo.12205391.

Members of the Darwin Tree of Life Consortium are listed here: https://doi.org/10.5281/zenodo.4783558.
