# Supplementary material for: The genome sequence of the club-tailed millipede, Cylindroiulus punctatus (Leach, 1816)
Source: Wellcome Open Res. 2025 May 20;10:256. [Version 1] doi: 10.12688/wellcomeopenres.24251.1 (PMC12181768; doi:10.12688/wellcomeopenres.24251.1)
Supplement: Peer review history

# Reviewer report: Reviewer response for version 1

**DOI:** 10.21956/wellcomeopenres.26748.r124737

**Published:** 2025-06-20

**Authors:** Wai Lok So

**Affiliations:** 1 The Chinese University of Hong Kong, Hong Kong, China

The authors have generated a high quality genome of a widespread British millipede, *Cylindroiulus punctatus*, with PacBio sequencing and further polished with Hi-C scaffolds. The assembled genome consists of mainly 7 chromosomal scaffolds, together with a ~19kb mitochondrial genome. The method used are sound and standard. There are only a few concerns on the information of the sample used for the experiment that has to be addressed, as stated below:

 - Are all the specimen collected adults?

 - Can the sex of the samples used for PacBio and Hi-C be identified?

 - What are the stage and sex of the sample use for RNA sequencing?

Are sufficient details of methods and materials provided to allow replication by others?

Yes

Is the rationale for creating the dataset(s) clearly described?

Yes

Are the datasets clearly presented in a useable and accessible format?

Yes

Are the protocols appropriate and is the work technically sound?

Yes

Reviewer Expertise:

myriapods, evolution, genome, biodiversity

I confirm that I have read this submission and believe that I have an appropriate level of expertise to confirm that it is of an acceptable scientific standard.

# Reviewer report: Reviewer response for version 1

**DOI:** 10.21956/wellcomeopenres.26748.r124262

**Published:** 2025-06-16

**Authors:** Zhi-Sheng Zhang

**Affiliations:** 1 Southwest University, Chongqing, China

This manuscript present a chromosome level genome assembly of a female club-tailed millipede *Cylindroiulus punctatus,* and the genome sequence with a total length of 354.09 Mb,  with 7 chromosomes. These genome is important for further research of millipede.  The dataset has been clearly described and presented in a useable and accessible format.

Are sufficient details of methods and materials provided to allow replication by others?

Yes

Is the rationale for creating the dataset(s) clearly described?

Yes

Are the datasets clearly presented in a useable and accessible format?

Yes

Are the protocols appropriate and is the work technically sound?

Yes

Reviewer Expertise:

genome, RNA, evolution.

I confirm that I have read this submission and believe that I have an appropriate level of expertise to confirm that it is of an acceptable scientific standard.
